# Two successful cases of DIEP flaps for breast reconstruction in patients with Factor V Leiden

**DOI:** 10.1093/jscr/rjy231

**Published:** 2018-09-06

**Authors:** Dmitry Zavlin, Ashley J Steinberg, Vishwanath Chegireddy, Aldona J Spiegel

**Affiliations:** Institute for Reconstructive Surgery, Houston Methodist Hospital, Weill Cornell Medicine, Houston, TX, USA

## Abstract

Factor V Leiden (FVL) is the most common inherited hypercoagulable condition. It is a genetic disorder caused by a missense mutation that prevents inactivation of Factor V in the clotting cascade, leading to overproduction of thrombin and excess clotting. This pathophysiological process is especially unfavorable in patients undergoing free tissue transfer. Many authors have noted a propensity for both venous and arterial thrombosis leading to partial or complete flap loss. To date, there have been no published reports of patients with FVL undergoing deep inferior epigastric perforator flap reconstruction without flap complications. Here, the authors present two cases of successful free tissue transfer for breast reconstruction in patients with diagnosed FVL. The perioperative thromboelastography lab values are evaluated to help guide anticoagulation regimen for these high-risk procedures.

## INTRODUCTION

Microsurgical procedures performed by skilled surgeons have shown a relatively low complication rate. In those rare instances of adverse events, the most common indication for reoperation in microvascular surgery is pedicle thrombosis [1]. Most microsurgical breast reconstructions are therefore performed in healthy patients with minimal risk factors for flap failure. Patients with unrecognized hypercoagulability are at risk of free flap failure independent of patient selection or technical errors [2]. For these reasons, most plastics surgeons avoid using microsurgical techniques on patients with an established diagnosis of hypercoagulability [3]. Factor V Leiden (FVL) is considered a relative contraindication to microsurgical reconstruction and many surgeons will perform implant based or pedicled flap reconstruction with such an established diagnosis. Currently, there are no reports of uneventful free flap breast reconstruction in the setting of FVL. The authors present their successful experience with two FVL patients who underwent deep inferior epigastric perforator (DIEP) flaps for breast reconstruction without any postoperative complications while using TEG for coagulation monitoring.

## CASE REPORTS

**Figure 1:**
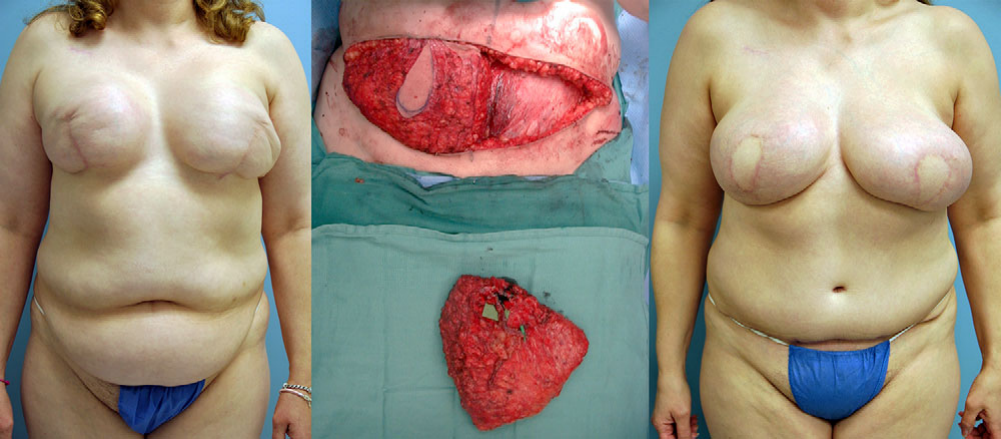
Pre, intra and 2-year postoperative images of patient #1 (left to right).

The first case describes a 48-year-old female who had an established diagnosis of heterozygous FVL with a positive family history and was diagnosed with left breast cancer in October 2010. She presented to our institution in November 2013 with painful capsular contracture from prior implant-based reconstruction and a desire for bilateral autologous reconstruction. After detailed counseling regarding her operative risks, she underwent bilateral implant removal, capsulectomy and bilateral sensate DIEP flaps. There were no significant perioperative adverse events. The patient received 3000 IU of intravenous unfractionated heparin (UFH) after both sets of anastomoses were performed. At her 2-year follow-up at our institution in early 2016, the patient was in good health with her flaps sensate and well-perfused (Fig. 1).

**Figure 2:**
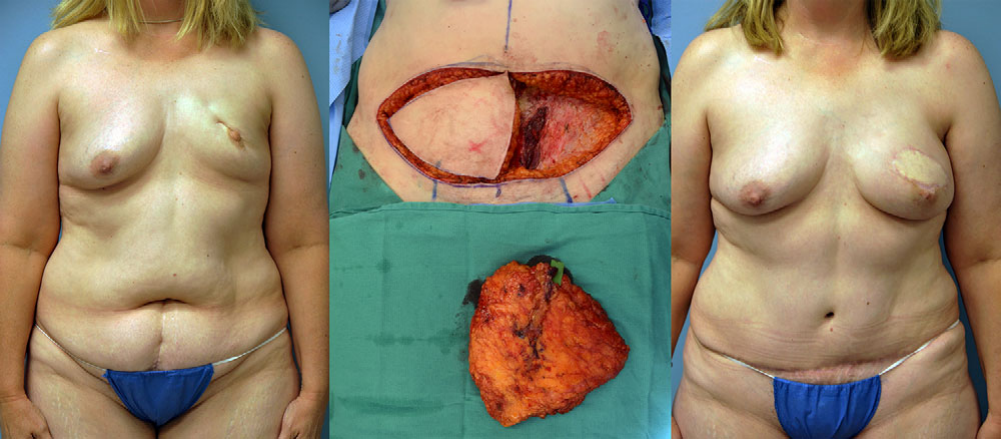
Pre, intra and 1-year postoperative images of patient #2 (left to right).

The second case involved a 49-year-old female who was diagnosed with infiltrating ductal carcinoma in 2010. After initial lumpectomy and subsequent chemoradiation, she presented in 2016 expressing desire for mastectomy of the left breast with autologous breast reconstruction. Her history was significant for heterozygous FVL and a previous lower extremity deep venous thrombosis which required 2 years of warfarin therapy. She underwent left completion mastectomy with neurotized DIEP flap reconstruction. There was clotting noted intraoperatively prior to performing the anastomosis, and the decision was made to irrigate the vessels with tissue plasminogen activator solution. The patient was given an intravenous dose of 3000 units of UFH. At her 1-year follow-up in August 2017, her flaps and abdominal scar were inconspicuous and the patient was awaiting her symmetry breast revision procedures (Fig. 2).

All lab values of both patients are displayed in Table 1 and their medications in Table 2. The TEG® 5000 Thromboelastograph® Hemostasis System (Haemonectics Corporation, Braintree, MA) was the device performing all TEG tests. Additionally, the thrombocyte count, prothrombin time (PT) and activated partial thromboplastin time (aPTT) were obtained.

**Table 1:** Lab results.

Variable	FVL case 1	FVL case 2	Reference range^a^
Baseline			
Thrombocytes (1000/ml)	342	267	150–400
aPTT (s)	31.0	26.9	23.0–34.0
PT (s)	12.6	11.8	12.0–15.0
TEG-R (min)	5.6	6.0	2.5–7.5
TEG-K (min)	1.3	1.3	0.8–2.8
TEG-SP (min)	5.2	5.7	N/A
TEG-G (dyn/cm^2^)	10 671	10 976	4600–10 900
Intraop			
Thrombocytes, 1000/ml	274	197	
aPTT (s)	50.5	28.1	
PT (s)	14.3	13.5	
TEG-R (min)	6.8	5.4	
TEG-K (min)	1.6	1.8	
TEG-SP (min)	6.4	4.8	
TEG-G (dyn/cm^2^)	6792	7517	
POD1			
Thrombocytes (1000/ml)	248	196	
TEG-R (min)	4.4	3.7	
TEG-K (min)	1.2	1.2	
TEG-SP (min)	4.1	2.8	
TEG-G (dyn/cm^2^)	9659	9669	
POD2			
Thrombocytes (1000/ml)	228	183	
aPTT (s)	38.3	27.0	
PT (s)	14.5	13.3	
TEG-R (min)	5.2	5.0	
TEG-K (min)	1.2	1.4	
TEG-SP (min)	4.8	4.4	
TEG-G (dyn/cm^2^)	10 600	11 145	

^a^At our institution.

Baseline: 2–3 weeks preoperatively.

POD0: intraoperatively, shortly after UFH administration.

POD1: postoperative Day 1.

POD2: postoperative Day 2.

**Table 2:** Patient medication.

Drug	FVL case 1	FVL case 2
Aspirin	81 mg daily^a^	81 mg daily for 2 weeks
Postoperative enoxaparin	40 mg daily until hematology consult	40 mg daily until hematology consult
Intraoperative UFH	3000 IU, single dose	3000 IU, single dose
Postoperative UFH	32 h infusion	48 h infusion
Warfarin	–	Discontinued^a^

^a^Home medication.

## DISCUSSION

Advances in microsurgical technique have led to extremely low flap failure rates, with an upwards of 99% flap survival rate for breast reconstruction [4]. Free tissue transfer has become the mainstay of treatment for many soft tissue deficits, including post-mastectomy breast reconstruction. Coagulopathy is one of the most common causes of flap failure, second only to technical error [1]. The majority of plastic surgeons are therefore hesitant about performing microvascular procedures in patients with an established hypercoagulability diagnosis [3]. Wang *et al.* [5] retrospectively analyzed all patients with hypercoagulability diagnoses as well as patients with prior thrombotic events who underwent free tissue transfer and found an alarming 15.5% rate of flap loss in these patients. Several other authors have shown a high rate of complications in patients with FVL after DIEP flap reconstruction [2, 6–8]. There are no similar reports in the literature of DIEP flap reconstruction on patients with established FVL without postoperative complications—neither systemic, nor localized to the surgical site. Furthermore, there is a complete lack of reports on homozygous FVL patients undergoing free flaps.

Anticoagulation has noted to be beneficial in patients who have a history of thrombotic events or underlying hypercoagulable state [9]. In our practice, thromboelastography (TEG) is used in every patient to guide our use of anticoagulation both intraoperatively and postoperatively. Our intraoperative UFH use is dictated by preoperative TEG values [10].

In both of the presented cases, the patients had a prior diagnosis of heterozygous FVL. These patients underwent our algorithm with TEG, standardized intraoperative coagulation with UFH and postoperative anticoagulation with UFH, enoxaparin and aspirin [10]. The critical time where most flap failures occur is intraoperatively or in the immediate postoperative phase (0–72 h) [1]. Therefore, the TEG was used during this time period to monitor patient’s hypercoagulability and prevent flap thrombosis. Both patients received 3000 units of UFH intraoperatively based on borderline high preoperative TEG values (Table 1). In contrast, Handschin *et al.* [8] did not begin UFH administration until noticing an ischemic flap when the arterial anastomosis was already thrombosed. Khansa *et al.* [6] did not use any intravenous UFH in their two cases of FVL and flap failure. In our experience, the administration of intravenous UFH (half-life: 30 min) during pedicle anastomosis is an integral step to ensure optimal coagulation and successful microvascular breast reconstruction.

There is currently no established protocol, let alone any guidelines for patients with FVL or any other hypercoagulability disorders undergoing microvascular tissue transfer. Nevertheless, some authors do consider these disorders as relative contraindications for free flap surgery [5], meaning that the patients need to be made of aware of the increased hazards. In order to remain on the side of safety, the use of pre and postoperative anticoagulation regimens were altered and medications were dosed based on the primary surgeon’s experience. Both cases presented had no short or long term flap complications, suggesting that with proper perioperative management, patients with FVL can undergo microsurgical breast reconstruction with favorable results.

In conclusion, hypercoagulability is a well-known risk factor for complications in microvascular surgery leading many reconstructive surgeons to seek alternatives to complex microsurgical breast reconstruction in patients with an established diagnosis. In experienced hands, free flap breast reconstruction can be performed in these challenging conditions if the risks are outlined in detail and the patients are adequately treated and monitored in the perioperative period. Our proactive management led to no complications in three DIEP flaps.

## CONFLICTS OF INTEREST, FINANCIAL DISCLOSURES AND SOURCE OF FUNDING

None of the authors, nor their close family members, have a financial interest in any of the products, devices or drugs mentioned in this manuscript. Furthermore, the authors declare that no commercial associations or financial disclosures exist that might pose or create a conflict of interest with information presented in this manuscript. No funding was received for the work presented in this manuscript.

## ETHICAL CONSIDERATIONS

The work described in this article was approved by our institutional review board (Protocol number 00011704: ‘Observational Research in the Department of Plastic and Reconstructive Surgery’). The authors adhered to the Declaration of Helsinki at all times.

## References

[1] Bui DT, Cordeiro PG, Hu QY, Disa JJ, Pusic A, Mehrara BJ Free flap reexploration: indications, treatment, and outcomes in 1193 free flaps. Plast Reconstr Surg 2007;119:2092–2100. 17519706 10.1097/01.prs.0000260598.24376.e1

[2] Davison SP, Kessler CM, Al-Attar A Microvascular free flap failure caused by unrecognized hypercoagulability. Plast Reconstr Surg 2009;124:490–5. 19644264 10.1097/PRS.0b013e3181adcf35

[3] Sezgin B, Ayhan S, Tuncer S, Sencan A, Aral M Hypercoagulability in microvascular breast reconstruction: an algorithmic approach for an underestimated situation. J Reconstr Microsurg 2012;28:515–20. 22744893 10.1055/s-0032-1315771

[4] Gill PS, Hunt JP, Guerra AB, Dellacroce FJ, Sullivan SK, Boraski J, et al A 10-year retrospective review of 758 DIEP flaps for breast reconstruction. Plast Reconstr Surg 2004;113:1153–60. 15083015 10.1097/01.prs.0000110328.47206.50

[5] Wang TY, Serletti JM, Cuker A, McGrath J, Low DW, Kovach SJ, et al Free tissue transfer in the hypercoagulable patient: a review of 58 flaps. Plast Reconstr Surg 2012;129:443–53. 21987047 10.1097/PRS.0b013e31823aec4d

[6] Khansa I, Colakoglu S, Tomich DC, Nguyen MD, Lee BT Factor V Leiden associated with flap loss in microsurgical breast reconstruction. Microsurgery 2011;31:409–12. 21503971 10.1002/micr.20879

[7] McAllister P, Teo I, Chin K, Makubate B, Alexander Munnoch D Bilateral breast reconstruction with abdominal free flaps: a single centre, single surgeon retrospective review of 55 consecutive patients. Plast Surg Int 2016;2016:6085624. 27504200 10.1155/2016/6085624 PMC4967676

[8] Handschin AE, Guggenheim M, Calcagni M, Kunzi W, Giovanoli P Factor V Leiden mutation and thrombotic occlusion of microsurgical anastomosis after free TRAM flap. Clin Appl Thromb Hemost 2010;16:199–203. 19022796 10.1177/1076029608325546

[9] Pederson WC Clinical use of anticoagulants following free tissue transfer surgery. J Hand Surg Am 2008;33:1435–6. 18929217 10.1016/j.jhsa.2008.08.008

[10] Zavlin D, Jubbal KT, Agrawal N, Spiegel AJ Abstract: thromboelastography and intraoperative anticoagulation during reconstructive microsurgery. Plast Reconstr Surg Glob Open 2017;5:129.

